# Structure of cortical network activity across natural wake and sleep states in mice

**DOI:** 10.1371/journal.pone.0233561

**Published:** 2020-05-29

**Authors:** Kaoru Ohyama, Takeshi Kanda, Takehiro Miyazaki, Natsuko Tsujino, Ryo Ishii, Yukiko Ishikawa, Hiroki Muramoto, Francois Grenier, Yuichi Makino, Thomas J. McHugh, Masashi Yanagisawa, Robert W. Greene, Kaspar E. Vogt

**Affiliations:** 1 WPI-IIIS, International Institute for Integrative Sleep Medicine, University of Tsukuba, Tsukuba-shi, Ibaraki, Japan; 2 Laboratory for Circuit and Behavioral Physiology, RIKEN Brain Science Institute, Wako-shi Saitama, Japan; 3 Peter O'Donnell Brain Research Institute, UT Southwestern Medical Center, Dallas, TX, United States of America; Sorbonne Universite UFR de Biologie, FRANCE

## Abstract

Cortical neurons fire intermittently and synchronously during non-rapid eye movement sleep (NREMS), in which active and silent periods are referred to as ON and OFF periods, respectively. Neuronal firing rates during ON periods (NREMS-ON-activity) are similar to those of wakefulness (W-activity), raising the possibility that NREMS-ON neuronal-activity is fragmented W-activity. To test this, we investigated the patterning and organization of cortical spike trains and of spike ensembles in neuronal networks using extracellular recordings in mice. Firing rates of neurons during NREMS-ON and W were similar, but showed enhanced bursting in NREMS with no apparent preference in occurrence, relative to the beginning or end of the on-state. Additionally, there was an overall increase in the randomness of occurrence of sequences comprised of multi-neuron ensembles in NREMS recorded from tetrodes. In association with increased burst firing, somatic calcium transients were increased in NREMS. The increased calcium transients associated with bursting during NREM may activate calcium-dependent, cell-signaling pathways for sleep related cellular processes.

## Introduction

Behavior of cortical neurons in wake (W) is characterized by ongoing irregular action potential firing [1]. During non-rapid eye movement sleep (NREMS), cortical neurons synchronously transition between a depolarized (UP) state and a hyperpolarized (DOWN) state [2], which is thought to be associated with an active ON period and a silent OFF period at extracellular multi-unit activity level [3]. Activity of cortical neurons during NREMS-ON/UP periods (NREMS-ON-activity) is similar to that during W (W-activity), leading to the hypothesis that the unique firing pattern of cortical neurons during NREMS is characterized by the interruption of W-activity by NREMS-OFF/DOWN periods [4,5]. On the other hand, it is clear that NREMS is a quite distinct functional state from W. In particular, functional connectivity is altered during NREMS compared to during W [6,7,8], which may affect the organization of NREMS-ON-firing patterns of cortical units. High and low wake active cortical neurons show reduced and increased activity in NREMS respectively [9], indicating a shift in functional organization. There is, therefore, a possibility that NREMS-ON-activity is different from W-activity in a number of aspects. We focused on the structure of spike trains from individual neurons and on the organization of activity in ensembles of neurons in the cortex in association with calcium transients. We calculated entropy of individual spike trains, which is a measure of the predictability of a firing pattern [10]. Activity patterns in ensembles of neurons in part reflect their functional architecture. In the cortex, even non-specific activation of a local network can evoke ordered activity—for example through synfire chains [11]. The potential impact of weakened functional connectivity in NREMS [6,8] on ordered ensemble activity is unexplored.

Using multi-site single unit recordings and two-photon calcium imaging, we examined whether NREMS-ON-activity has distinct properties from W-activity and also elucidated differences between both states by focusing on firing pattern of single neurons, multi-neuron spike sequences, and intracellular calcium dynamics.

## Materials and methods

### Surgery, tetrode recording and histology

**Fig 1:**
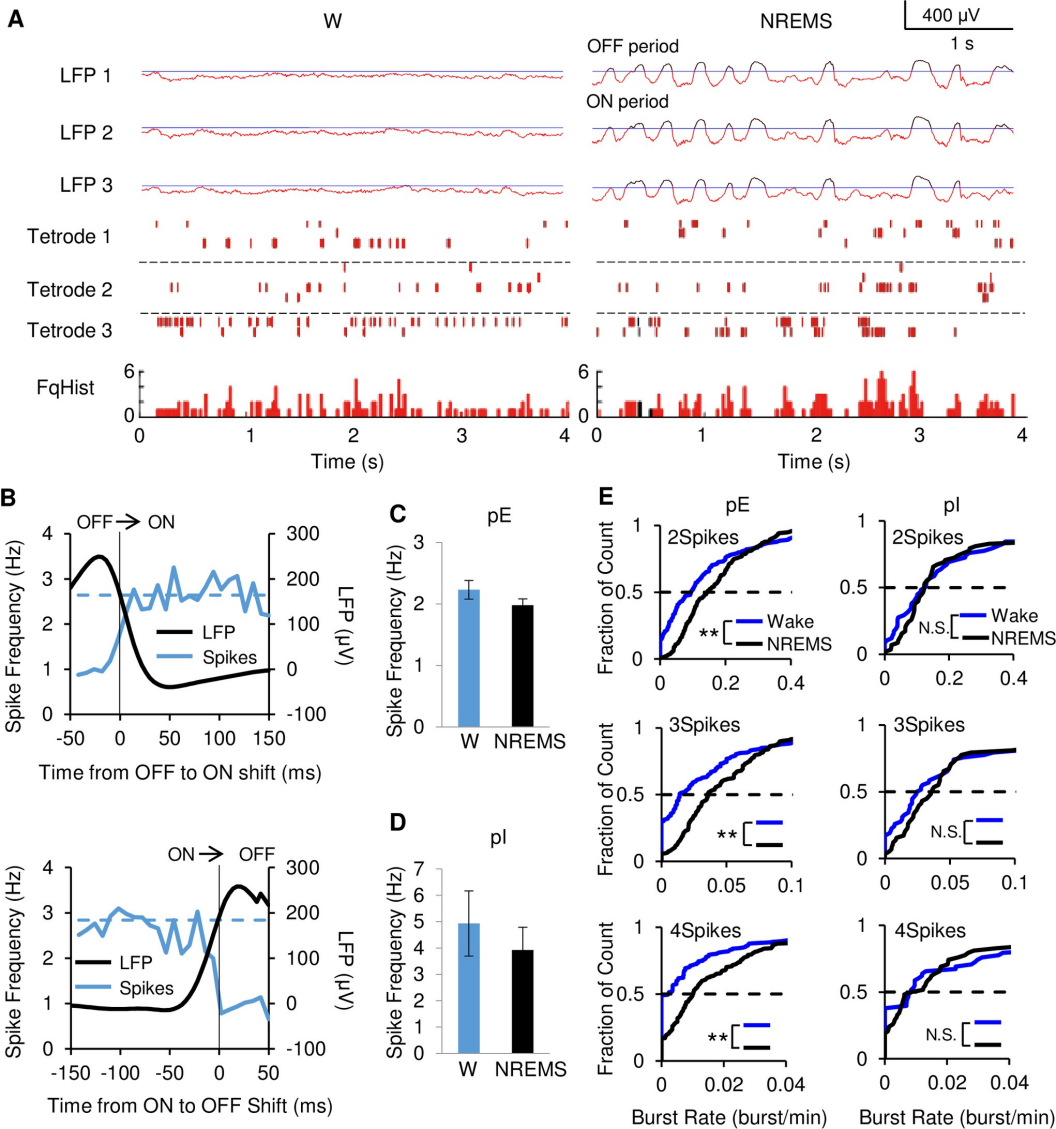
Small networks of cortical neurons change from irregular, tonic-like firing in W to rhythmic, ON/OFF burst-like activity in NREMS. **(A**) Simultaneous recordings from 3 tetrodes in deep layers of M1 cortex during W and NREMS. LFPs (top 3) and raster plots (middle 3) of the same 10 pE neurons during W (left) and NREMS (right). LFPs are color coded for ON periods (red) and OFF periods (black) and OFF period threshold (blue). The spikes in the raster plot are similarly color coded. Combined frequency histogram (FqHist) for all pE neurons is separately illustrated in the raster plots (Hz/25 ms) (bottom). During NREMS, the raster plots showed intermittent bursting and pauses during ON periods. In contrast, the combined frequency histogram showed sustained firing throughout the ON periods (indicating the random occurrence of individual neuronal bursting during an ON period) and no firing during OFF periods. **(B**) Triggered average of pE-neuron firing frequency and LFP amplitude at OFF-ON transition (top) and ON-OFF transition (bottom) for the population of pE neurons. Average firing frequency showed sustained constant increase in NREMS after transition from OFF to ON period, despite increased bursting of individual pE neurons in NREMS shown in (E). LFP (Black; right Y axis) correlated with firing frequency (Blue; Hz in 10 ms bins, left Y axis) plot during the NREMS OFF to ON transition (top) and ON to OFF transition (bottom). Average W firing frequency of the same neuron population is shown as dashed line. For OFF to ON transitions firing before transition was 0.99±0.08 Hz increasing to 2.6±0.18 Hz after transition (N = 284 transitions, p<0.01, t = -11.79, paired Student’s t-test). For ON to OFF transitions firing before transition was 2.5±0.29 Hz decreasing to 0.98±0.09 after the transition (N = 253 transitions, p<0.01, t = 6.53, paired Student's t-test). Firing during a 40 ms window at the beginning and the end of the ON period was not different (p = 0.85, t = 0.97, Student's t-test). **(C**) Average firing frequency throughout W and throughout NREMS for individual pE neurons. **(D**) Average firing frequency throughout W and throughout NREMS for individual pI neurons. **(E**) Cumulative histogram of the number of bursts per minute (Burst Rate) for 212 pE neurons (left) and 58 pI neurons (right). Plots for burst lengths of two (2Spikes), three (3Spikes) and four (4Spikes) spikes per burst. W (blue line) and NREMS (black line). Bursts are two or more spikes with interspike intervals<15 ms. Burst rates increased significantly from W to NREMS for pE (p < 0.01, Wilcoxon related samples signed rank test), but not for pI (p = 0.837 in 2Spikes, p = 0.118 in 3Spikes, p = 0.985 in 4Spikes, Wilcoxon related samples signed rank test). Double asterisks indicate p-value below 0.01 (Wilcoxon related samples signed rank test).

Tetrode recordings allow reliable identification of extracellular activity from individual neurons (or units) in small networks surrounding the tetrode tip, together with the recording local field potentials (LFPs) that reflect summed, mostly synaptic, activity in a slightly larger volume of tissue (Figs 1A and S1A). All experimental procedures were carried out in accordance with local and national regulations and after approval by the animal care and use committee of the University of Tsukuba. Seven male C57BL/6 mice (Jackson Laboratory; 3–6 months old) were employed for in vivo electrophysiology. Under isoflurane anesthesia (4% for induction and 1–2% for maintenance), nichrome tetrodes diameter of 14 μm (KANTHAL Precision Technology), screws, and wires for monitoring brain and muscle activity were implanted. Cortical region for inserting tetrodes were exposed by craniotomy. Three to four tetrodes were closely positioned at each recording site with tetrode spacing of 300 μm using a microdrive consisting of seven independently adjustable tetrodes. Target regions were the primary motor cortex (1.4 mm anterior and 2 mm lateral in right and/or left from bregma, 0.5–1.5 mm depth, Allen brain atlas) or the primary somatosensory cortex (0.9 mm posterior and 3 mm right-lateral from bregma, 0.5–1 mm depth, Allen brain atlas). One tetrode was inserted into the ventral hippocampal commissure (0.8 mm posterior and 0.8 mm right-lateral from bregma, 2.4 mm depth, Allen brain atlas) as reference electrode. Two stainless screws were implanted in the skull for monitoring surface EEG and flexible wires (Cooner wire) were implanted into the right and left neck muscles for monitoring EMG. A ground screw was implanted midsagitally in the skull over the cerebellum and additional screws were used for anchoring the recording microdrive (S2A Fig).

After two weeks of recovery, mice were habituated to the recording situation. Data acquisition and online spike detection were done using a 32-ch Digital Lynx 4SX system and Cheetah software (Neuralynx). Recordings were started as soon as at least one tetrode from one region showed a good S/N ratio for spike detection. Spike data was digitized at 32 kHz and spike waveforms were band pass filtered between 0.6–6 kHz. For LFP-, EEG-, and EMG-recordings, data was sampled at 250 Hz and high pass filtered at 0.1 Hz for LFP/EEG and 10 Hz for EMG. LFPs were recorded for each tetrode using one of the four tetrode channels. There was no significant difference in the location of peak delta power between these LFPs and surface EEG recordings (S2B–S2D Fig).

One EEG screw was used as reference for the other EEG screw. For EMG recording, the right (left) EMG wire was used as reference of left (right) EMG wire. Spike sorting was performed offline using Spikesort3D software (Neuralynx). Spikes were separated and clustered using peak amplitude, energy and waveform. To avoid clusters containing spikes from different neurons or artifact, clusters with interspike intervals less than 1 ms were removed from the analysis, considering the refractory period of a single neuron. For quantitative criteria of good separation of cluster from noise or the neighboring cluster, clusters with Isolation Distance less than 20 were discarded [12,13]. Clusters that contained fewer than 75 spikes were also discarded [12].

### Identification of putative pyramidal neurons and interneurons

Neurons were classified according to spike waveforms duration [14]. Units with a spike half-width shorter than 250 μs were classified as putative interneurons (pI neurons), while units with longer spike half-width were classified as putative excitatory neurons (pE neurons) (S3E Fig).

### Sleep score and vigilance state categorization

Vigilance states were identified according to surface EEG and neck muscle EMG recordings using a sleep scoring software written in Matlab [15] and results were manually checked, to verify that states were assigned correctly (S4 Fig). Standard criteria were used to classify vigilance states into waking, NREM sleep and REM sleep respectively at 4s epochs. Waking was characterized by low amplitude, high frequency activity in EEG and LFP recordings (Fig 1A left) and EMG signals indicative of neck muscle activity. NREM sleep was characterized by high-amplitude, low-frequency activity in EEG and LFP (Fig 1A right) and neck muscle EMG indicating steady low muscle tone. REM sleep was characterized by high-frequency, low-amplitude EEG and LFP signals accompanied by neck muscle atonia. Mean (+/- SEM) episode durations were 99 +/-16 s for wake, 146 +/-14 s for NREMS and 69+/-10 s for REMS.

### LFP ON-OFF period classification

The mean value of the LFP was determined for 4 s windows. In addition, the signal was filtered post-hoc in Matlab, with a band pass filter in the gamma band (7-pole Bessel filter with a 30–100 Hz pass band) and the RMS value of the mean-subtracted gamma oscillation was determined for this 4 s window. LFP values that were above the mean plus five times the gamma band RMS value were considered OFF period, whereas all other values were considered ON period. This method results in very few W-OFF periods and clear ON-OFF transitions in NREMS (S1A Fig, Fig 1A). To calculate LFP-triggered spike rates, the LFP was low-pass filtered at 5 Hz using a 7 pole Bessel filter in Matlab using the zero-phase shift 'filtfilt' function. Transitions of the filtered signal from above ON-period-threshold to below were considered as OFF-ON transitions and vice-versa. To detect spurious correlations between LFP and spike activity we repeated the same procedure after randomly shifting spikes by ± 1s; under these conditions no significant change in firing was observed across LFP ON-OFF or OFF-ON transitions (data not shown).

### Burst analysis

Bursts were defined as sequences of two or more spikes from the same unit with interspike intervals shorter than 15 ms [9,16]. Sequences of spikes that fulfilled this criterion were grouped into doublets, triplets, quadruplets and quintuplets according to the number of spikes in the burst.

### Entropy analysis

The best interpretation of Shannon information entropy in the context of this work is the orderliness or predictability of a distribution of events. Entropy is high if the events are not orderly and therefore unpredictable. Entropy is low if events are highly ordered and therefore more predictable. We have analyzed two different event distributions: First we analyzed the distribution of spike patterns from individual neurons, this measures how predictably a single neuron fires in time. Second, we analyzed the distribution of spike sequences in an ensemble of simultaneously recorded neurons, this measures the orderliness and predictability of how neurons fire one after another in sequence.

### Individual entropy rate

**Fig 2:**
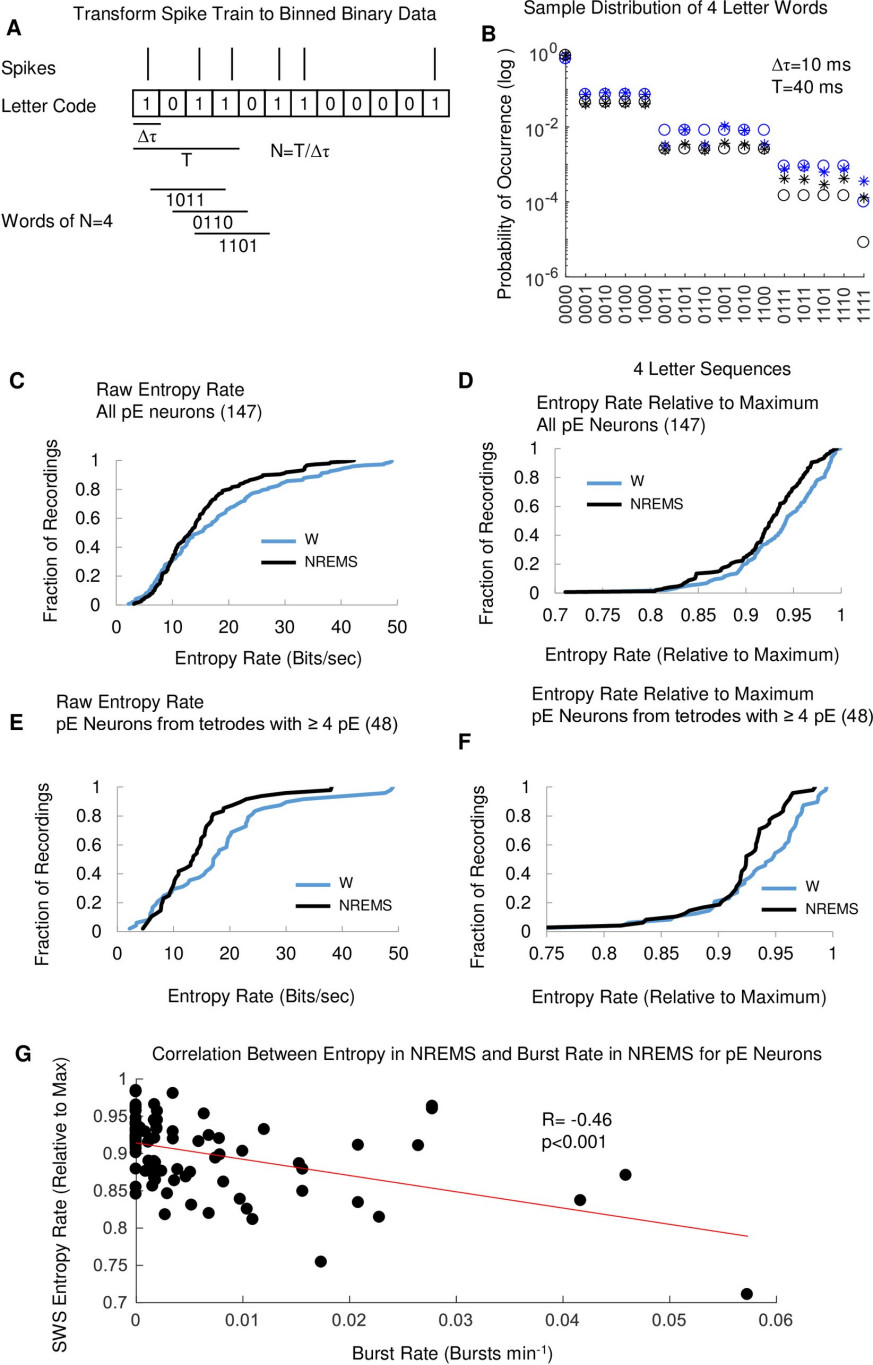
Single-neuron spike trains exhibit lower individual entropy rates in NREMS compared to W. **(A)** Schematic of the transformation of spike trains into a binned binary letter sequences and formation of N = four letter words. Bin duration is Δτ, word duration is T. **(B**) Examples of four-letter word distribution (0000 to 1111) in W (blue) and NREMS (black) from one M1 neuron recorded in W and NREMS. Stars show the actual occurrence probabilities and circles the calculated probabilities from the firing frequency (see Methods for definition of occurrence and calculated probability). **(C**) Individual entropy rate [(entropy for all words of duration T)/T] for pE neurons in W and NREMS for bin size 10 ms and word length 4. Entropy rate was significantly lower in NREMS (14.9±0.7 Bits/sec) compared to W (17.8±1.0 Bits/sec), (N = 147, p<0.001, Wilcoxon related samples signed rank test). **(D**) Entropy rate relative to maximal entropy rate in W and NREMS (see Methods). Relative NREMS entropy rate (0.92±0.004) was also significantly lower compared to W (0.94±0.004) (N = 147, p<0.001, Wilcoxon related samples signed rank test). **(E & F**) Same data as in C&D, but for pE neurons from tetrodes with four simultaneously recorded pE neurons. Both the entropy rate and the rate relative to maximum were lower in NREMS (14.2±1.1 Bits/sec and 0.92±0.006) compared to W (18.3±1.7 Bits/sec and 0.93±0.007) (N = 48, p<0.001 and p = 0.012, Wilcoxon related samples signed rank test). **(G**) Scatter plot of relative entropy rate in NREMS versus burst rate (black dots) for pE neurons. Red line shows linear fit to the data. Increased burst rates negatively correlated with relative entropy in NREMS. Pearson's correlation coefficients (R) and p values are indicated on the graphs (N = 105).

The entropy and entropy rates of spike trains from single neurons were calculated by converting spike time-points into time-binned binary patterns (Fig 2A) [10,17]. Each bin of duration Δτ represents a letter (1 or 0; 1 if one or more spikes occurs during the bin, otherwise, 0) and N consecutive letters form words of length T. Words with N (T/ Δτ) number of bins form a distribution of distinct words (D) with 2^N^ members (i). The observed patterns are tallied and the probability of occurrence of a given pattern (p_i_) is calculated by dividing their occurrence (Oi) by the total number of observed words of T length (O_i_/O_total_; Fig 2B). Each observed word contributes to the total entropy a of the distribution D as calculated according to Shannon's formula [17].

$${Entropy}_{D}=-\sum_{i=1}^{2^{N}}p_{i}\log_{2}p_{i}.$$ (Eq 1)

The individual entropy rate is calculated by dividing the entropy by the duration of a word (T).

The observed entropy rate was compared to the theoretical maximal entropy rate of the neuron. Theoretical maximal entropy Entropy_max_ results from a completely random occurrence of spikes. It is calculated by first obtaining the probability of firing (p_sp_) during Δτ (observed number of spikes/total number of Δτ in observation period) and then calculating the probability of occurrence of all the words of N length. Accordingly

$${Entropy}_{max}=-\sum_{i=1}^{N}C_{N}^{i}{p_{sp}}^{i}{{(1-p}_{sp})}^{N-i}\log_{2}{{(p}_{sp}}^{i}{{(1-p}_{sp})}^{N-i})$$ (Eq 2)

with

$$C_{N}^{i}=\frac{N!}{(N-1)!i!}$$

the binomial coefficient for i ones in N bins [10]. The maximal entropy rate was again calculated by dividing the entropy value by the duration T [17]. The percentage of spikes that fell onto the same bin was low in both W and NREMS (3.3 +/- 0.4% in W and 2.4 +/- 0.24% in NREMS, N = 48, p = 0.17, Kruskal-Wallis test).

### Ensemble entropy

**Fig 3:**
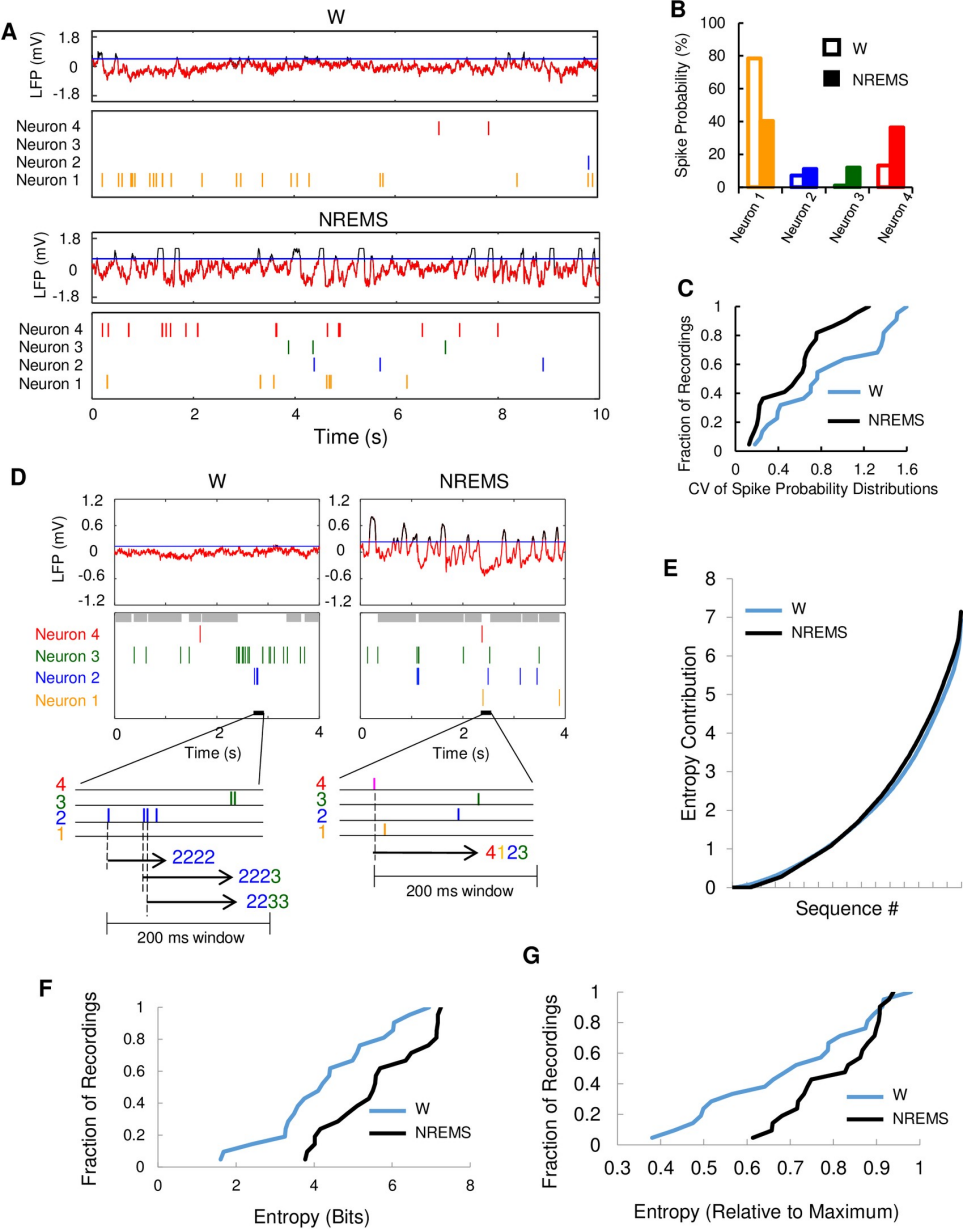
Neuronal ensembles in local cortical region show increased entropy of spike patterns in NREMS compared to W. **(A**) Sample firing patterns from neurons recorded from one M1 tetrode during W and NREMS; LFP (top) and raster plot (below) of 4 identified pE neurons in W and NREMS. LFP ON period (red), OFF period (black) and OFF period threshold (blue). **(B**) Probability distribution of firing frequency for each of the 4 neurons (shown in A; see Methods) recorded within local cortical region in W (open bars), and NREMS (solid bars). **(C**) Probability distributions of firing frequency for neurons in small cortical networks were less variable in NREMS compared to W. Cumulative histogram of the coefficient of variation (CV) of probability distributions (W, 0.87±0.1; NREMS, 0.56±0.07, N = 22, p<0.001 Wilcoxon related samples signed rank test). For each tetrode four or more identified neurons were recorded in both W (blue) and NREMS (black). **(D**) Sample sequence measurement from one S1 tetrode recording in W and NREMS with LFP (top) and raster plot (middle). Enlarged section (bottom) illustrates spike sequences computation. Grey bars in raster plot illustrate firing pauses > 200 ms. **(E**) Sample plot of cumulative contribution to the ensemble entropy for a 256 (4 neurons ^ 4 sequences) element spike pattern distribution. Sequences were collected from the same set of neurons in W and NREMS and ordered by their size of the contribution to entropy (-p_i_log_2_(p_i_) (see Methods). **(F**) Cumulative histogram for the ensemble entropy of 4 spike sequences in W and NREMS for sets of 4 simultaneously recorded neurons. 4.2 ±0.3 Bits in W versus 4.5±0.3 Bits in NREMS (N = 21, p<0.001, Wilcoxon related samples signed rank test). **(G**) Cumulative histogram for ensemble entropy normalized to maximum ensemble entropy for the dataset in F—0.70±0.04 in W and 0.81 ±0.02 in NREMS (N = 21, p<0.006, Wilcoxon related samples signed rank test).

Recordings with four or more stably recorded pE neurons during both W and NREMS during a continuous recording session were used for analysis of spike-sequence entropy. Spikes were considered part of a sequence if they occurred within a time window shorter than 200 ms. Spikes could be part of several four-spike sequences. E.g. a spike sequence pattern such as 13423243234 would result in the sequences 1342, 3423, 4232, 2324, and so on (Fig 3A & 3D). If a sequence could not be completed within the 200 ms set time window the next spike after the gap would be chosen as a new starting point and sequence tallying would continue (Fig 3D). Spikes that were part of such sequences were counted for either W or NREMS. A given units’ relative probability of firing frequency was calculated as the number of spikes from this unit divided by the number of all spikes from identified units. Coefficients of variation (CV) were calculated for these probability distributions of firing frequency in both W and NREMS (Fig 3C).

The valid sequences were tabulated into their N^length^ possible distinct sequences (N = number of unique units; length = number of spikes in a sequence) and the probability of occurrence of a sequence (p_se_) was calculated by dividing the number of tallied sequences of a given type by the total number of sequences. The entropy of these spike sequence distributions was again calculated according to the Shannon’s formula

$$Entropy=-\sum_{se=1}^{N^{length}}p_{se}\log_{2}p_{se}$$ (Eq 3)

The probabilities of the theoretical random distribution were calculated by multiplying the individual probabilities of firing frequency of the involved units. For a tetrode recording with 4 identified unique units the probability p_se_ of e.g. the sequence 1324 is p_1324_ = p_1_*p_3_*p_2_*p_4_. The entropy of this theoretical random distribution was again calculated using Eq 3. The theoretical random distribution is affected by changes in spike probabilities and will typically differ between W and NREMS, but it is blind to spike sequence since it assumes complete randomness. Differences between the actually observed distribution and this theoretical random distribution are therefore only due to the ordering of spikes and not in their relative abundance. We expressed this difference as the ratio of actual ensemble sequence entropy to entropy of theoretical random distribution.

### Calcium imaging

#### Surgery

We used Vgat-tdTomato mice that were generated by crossing Vgat-ires-Cre mice (*Slc32a1*^*tm2(cre)Lowl*^/J, Jackson Laboratory stock number: 016962) [18] with Ai9 tdTomato reporter mice (B6.Cg-*Gt(ROSA)26Sor*^*tm9(CAG-tdTomato)Hze*^/J, Jackson Laboratory stock number: 007909) [19]. All Vgat-tdTomato mice were maintained on a C57BL/6 background and genotyped by polymerase chain reaction of tail DNA. In Vgat-tdTomato mice, tdTomato was expressed accurately and specifically in GABAergic neurons. Thus, tdTomato-negative and -positive neurons were defined as pE and pI neurons, respectively. In this study, only Ca^2+^ imaging data on pE were analyzed. Five adult (>20 weeks) female Vgat-tdTomato mice were prepared for chronic two-photon imaging. During surgery, the mice were anaesthetized with isoflurane. A craniotomy (~3 mm diameter) was made over the right somatosensory or motor cortex. AAV vectors encoding GCaMP5G (AAV2/8-hSyn-GCaMP5G-WPRE, >10^12^ genomic copies/μl) were injected at a depth of 300 μm into 2–3 sites (~150 nl each) at a rate of 10–15 μl/min with a microinejctor (BJ110 and BT200, BEX). After AAV vector injection, a glass coverslip coated with Lipidure (NOF Corporation) was placed on the dura, and the edge was glued to the skull with Superbond (Sun Medical). Screws were placed on the right frontal and parietal bones for EEG and flexible wires were inserted into the neck muscles for EMG recordings. A stainless-steel head plate was cemented to the skull with light cure resin cement (3M ESPE). The mice were returned to home cages after surgery.

#### Two-photon calcium imaging during NREMS and W

Head-restraint on an air-supported spherical treadmill [20] was used for imaging of cortical neurons in sleeping/waking mice. Following >14 days after AAV injection, mice were gradually acclimatized (> 5 days) to the recording system, by connecting them to the head restraint apparatus for 5 h (ZT3-ZT8, 12:00–17:00). Imaging was conducted from ZT3 to ZT8 with an upright two-photon laser-scanning microscope (Axio Examiner Z1/LSM780, Zeiss) based on a mode-locked Ti:sapphire laser at 910 nm (Maitai DeepSee, Spectra-Physics) and a water-immersion objective (W Plan Apochromat 20x/1.0 DIC D = 0.17 M27 75mm, Zeiss). GCaMP5G fluorescence was detected with a gallium arsenide phosphide photomultiplier tube through a 500–550 nm band-path filter. Ca^2+^ signals in neurons in layer 2/3 (150–250 μm form the dura) were imaged at 4 frames/s for somatosensory and 8 frames/s for motor cortex (128x256 pixels, 16 bit). EEG signals were amplified 40000x (20x and 2000x) and band-pass filtered between 0.5 and 500 Hz. EMG signals were amplified 4000x (20x and 200x) and band-pass filtered between 1.5 and 1000 Hz. EEG/EMG signals were digitalized at 2000 Hz with a 16-bit analog-to-digital converter (Digidata 1440A, Molecular Devices), and acquired with Clampex 10.3 software (Molecular Devices).

#### Image processing and data analysis

Image stacks were aligned to remove potential motion artifacts. Regions of interest (ROIs) were drawn on somata of GCaMP5G-expressing neurons. Average fluorescence signal within a ROI F was calculated following subtraction of background signal (average fluorescence signal of a cell-free region) for each frame with a homemade program in ImageJ (NIH). ΔF/F_0_ = (F-F_0_)/F_0_, where F_0_ is the time-dependent baseline (Jia et al. 2011). In this study, neuronal activity-dependent signal was defined as a transient with rapid rise in ΔF/F_0_ followed by exponential decay with a tau of >1 s. ROIs without the fast transients were excluded from the analysis. Peaks in the ΔF/F_0_ transients were detected automatically using Matlab peak detection algorithm using a 2 s window size.

#### Action potential-based model of calcium concentration

Each unit activity was modeled to cause a rise in calcium with a mono-exponential time constant tau_on and a mono-exponential decay time constant tau_off and amplitude A. We used data from a detailed study on action potential-evoked calcium transients in cortical neurons [21] to choose the parameters. In our standard model the amplitude A of a Ca^2+^ transient evoked by a single action potential was set to 400 nM, tau_on was 10 ms and tau_off 100 ms. To explore the effect of different time constants we also modeled calcium transients using 200 ms, 400 ms and 800 ms tau_off rates (S4 Fig). Transients started from- and decayed to a resting intracellular Ca^2+^ concentration of 200 nM. Overlapping transients were summed linearly.

### Data presentation, experimental design and statistical analysis

Larger datasets are mainly presented in the form of cumulative histograms, which include all data points relevant for the statistical analysis. Statistical tests are described in their respective sections. In 30 of 33 statistical analyses we used non-parametric tests, since normality could not be assumed. In 3 cases parametric tests were used due to large sample numbers and after tests for normality. Significance levels were set at p<0.05.

## Results

Tetrode recordings of identified simultaneous single unit activity were maintained across W and NREMS episodes, allowed testing of sequence activity of ensembles of neurons and firing patterns of individual neurons, in correlation with local field potentials (LFPs). In total, 313 single neurons were recorded from primary motor (M1) or primary somatosensory (S1) cortices using tetrode recordings (n = 155) across one or more episodes, each, of W and NREMS, in freely behaving mice (n = 7), results across recording areas were pooled. Neurons were classified as either putative excitatory neurons (pE neurons; 246/313, 79%) or putative inhibitory neurons (pI neurons; 67/313, 21%) based on their spike half-width [14] (S3E Fig). Altogether, pE (n = 246) and pI (n = 67) neurons were analyzed for individual firing pattern and rate in correlation with LFP.

### Neuronal activity during NREMS-ON periods compared to W

Neuronal firing rates during the ON period resemble W activity [4] and it was speculated that NREMS represented rhythmically interrupted W [4,5]. Our tetrode recordings provided an opportunity for comparison of individual neuronal firing patterns and rate during W to NREMS-ON periods. The relationship between NREMS LFPs and individual single unit activity was assessed by a time-locked alignment to multiple NREMS, large positive LFP transients (see Methods, S1 Fig & Fig 1), allowing an average of the unit activity with respect to the LFP (Fig 1B). This provided clear identification of distinct ON/OFF periods (S1A Fig & Fig 1A). During waking, W-OFF periods, classified by LFPs, occurred rarely (0.78+/- 0.08% of NREMS occurrence) (S1B Fig & Fig 1A) and were not analyzed. The average firing frequency in W was not significantly different from NREMS for all pE neurons (2.2+/-0.15 Hz in W and 2.0+/-0.10 Hz in NREMS, N = 246, p = 0.45, Wilcoxon related samples signed rank test) (Figs 1C and S3) or for pI neurons (4.9+/-1.2 Hz in W and 3.9+/-0.9 Hz in NREMS, N = 67, p = 0.50, Wilcoxon related samples signed rank test) (Figs 1D and S3).

During the NREMS-ON periods, the average pooled frequency for pE was constant (Fig 1B) with no indication of increased firing rate at the onset of the ON period nor decreased rate at the end of the ON period, as observed in urethane-anesthetized rats using extracellular electrode recordings [22] or in vivo calcium imaging [23].

Since firing rate does not necessarily take firing pattern of particular neurons across W and NREMS states into consideration, we next examined this to better determine the relationship of neuronal activity during ON periods compared to W.

### Increased bursting activity during NREMS-ON periods

During W, neocortical neurons can exhibit, to varying degrees, both tonic and bursting-like spiking patterns [9]. We quantified bursting by defining bursts as having 2 or more spikes with an interspike interval of less than 15 ms and found that cortical neurons had an intermittent firing burst pattern throughout the ON period, a pattern that pE, but not pI showed significantly less often during W (Fig 1E, S1 Table). Bursts occurred intermittently and showed a weak preference for the middle (S1C Fig), but not the beginning or end of ON periods. The bias for pE bursting during NREMS held for 2, 3 and 4 spike bursts (Fig 1E) and for interspike intervals of 5, 10 and 20 ms (S1 Table). Thus, although the firing rates of identified neurons are similar between W and NREMS-ON periods, the burst analysis revealed clear difference in the temporal pattern of firing.

### Lower entropy of single neuron spike pattern in NREMS compared to W

We transformed the spike patterns of individual neurons into binned binary sequences (Fig 2A) and calculated the individual entropy rate for these spike patterns in W and NREMS [17]. Individual entropy rates for pE were significantly lower in NREMS compared to W for all pE (Fig 2C). Individual entropy rates are highly sensitive to average firing rate, we therefore normalized the raw entropy rates to the maximal entropy rate for the given average firing rate in both W and NREMS [17]. The relative individual entropy rate was also significantly smaller in NREMS compared to W for all pE (Fig 2D). Significantly smaller individual entropy rates and relative entropy rates were also observed in recordings with multiple identified pE per tetrode (Fig 2E & 2F). Increased bursting in NREMS was significantly correlated with lower individual entropy in NREMS (Fig 2G). Thus, temporal firing patterns of individual pE neurons became more predictable, in association with a higher propensity for bursting.

### Greater ensemble entropy in networks of cortical neurons during NREMS compared to W

A recapitulation of W activity in NREMS predicts that individual neurons retain their relative firing rates across states. However, low activity neurons increase their firing rates and high activity neurons reduce activity in the transition from W to NREMS so that overall population firing rates are more homogenous (S3 Fig), consistent with previous observations in the neocortex [9,24]. This potentially indicates less organized ensemble firing patterns (greater ensemble entropy) as reflected by a significant decrease in the coefficient of variation (CV) of the relative spike probabilities between W and NREMS (Fig 3B & 3C).

The high temporal resolution of tetrode recordings enabled characterization of the unit firing sequences amongst four or more unit ensembles of neurons [25] (Fig 3D). We quantified the degree of randomness of four-spike sequences (Fig 3D) by calculating their Shannon entropy (see Methods) both in W and NREMS—each distinct sequence *i* contributing to the sum by -p_i_log2(p_i_) (Fig 3E). Ensemble spike sequences had significantly higher entropy in NREMS compared to W (Fig 3F). This entropy increase can be the result of more even spike probabilities among the contributing neurons, a higher randomness of their spike order, or both. To distinguish between these two possibilities, we calculated the ensemble entropy for completely random sequences using the measured spike probabilities in both W and NREMS (see Methods) and determined the ensemble entropy relative to this measure. Ensemble entropy relative to the maximum of random sequences was larger in NREMS compared to W (Fig 3G). The increased ensemble entropy in NREMS is therefore both due to the more even spike probabilities evidenced by the lower CV of the spike probability distribution of the contributing neurons and to a decreased orderliness of their sequences.

In summary, during NREMS compared to W, we observed spike patterns with significantly lower individual entropies in NREMS compared to W in pE neurons, correlated with their increased burst firing. At the same time, simultaneously recorded ensembles of pE neurons in local cortical networks showed more homogeneous spike probability distributions and greater ensemble entropy.

### Larger somatic calcium transients in cortical neurons during NREMS

**Fig 4:**
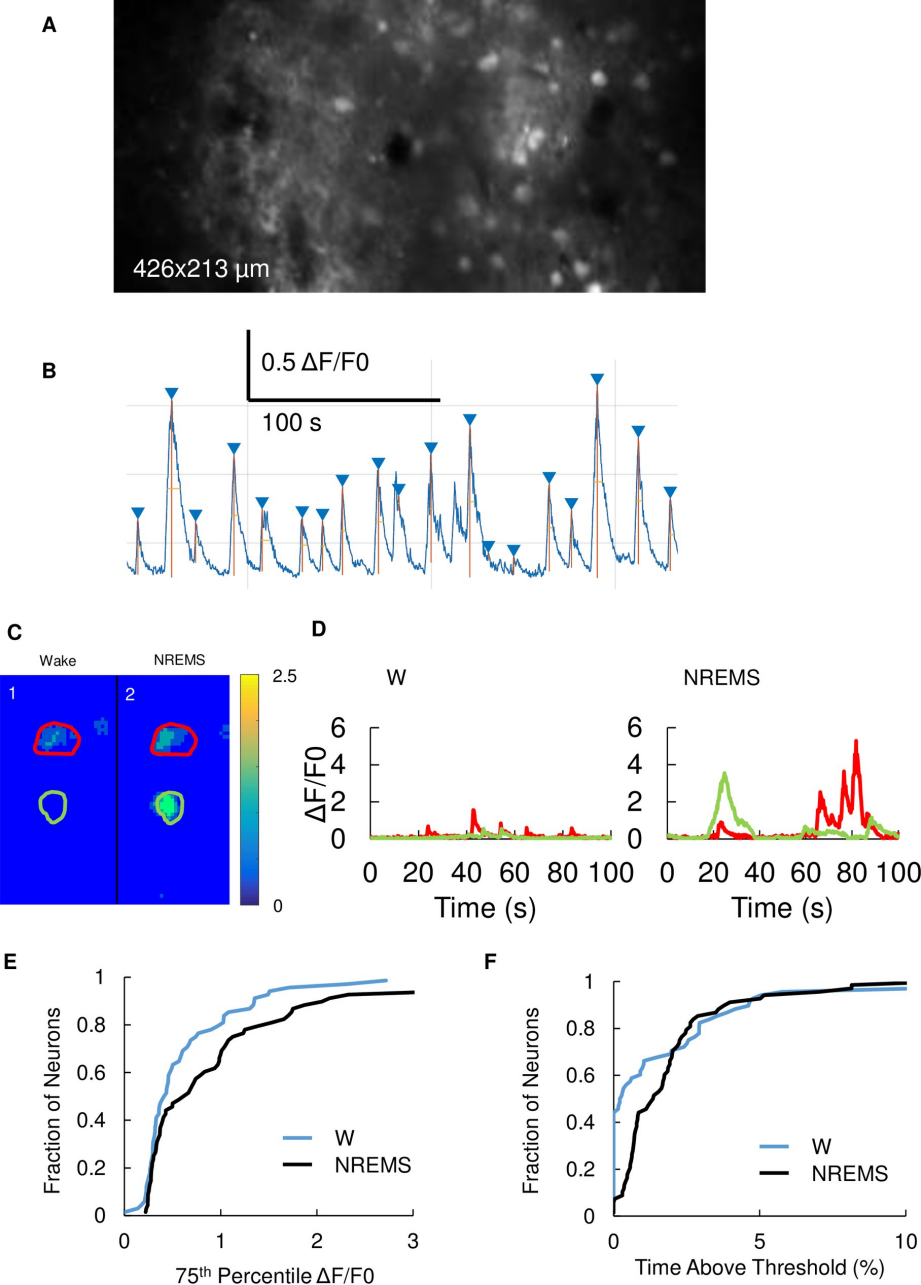
Larger intracellular Ca^2+^ transients observed in NREMS compared to W. **(A)** Representative two-photon image of GCaMP5G in S1 in-vivo. **(B**) Sample trace illustrating ΔF/F_0_ and peak detection. **(C**) Representative magnified pseudo-color images (53x106 μm) showing ΔF/F_0_ in W (1) and NREMS (2) in M1. Two images are of the same field of view. Adjacent color bar shows units in ΔF/F_0_. Two regions of interest (ROI) marked in red and green. **(D**) Sample ΔF/F0 traces in W (left) and NREMS (right). The red and green traces each show ΔF/F0 of the same color ROIs in C. **(E**) Ca^2+^ signal is lager in NREMS compared to W. Cumulative probability plot of the 75th percentile of peak ΔF/F_0_ transients in W (blue) and NREMS (black) which could be observed in both states during the same imaging session (N = 84 cells; W = 0.75±0.09; NREMS = 1.09±0.12, p<0.001, Wilcoxon related samples signed rank test). **(F**) Time above 75% of overall peak ΔF/F is longer in NREMS compared to W. Cumulative probability plot of time (as fraction of total time in state) during which ΔF/F values were above threshold in W (blue) and NREMS (black) for the same 84 pE (N = 84 cells; W = 1.7±0.3%; NREMS = 2.3±0.2%, p = 0.003, Wilcoxon related samples signed rank test).

We next examined different functions of NREMS-ON activity from W-activity. NREMS contributes memory consolidation [26], which is supported by activity-dependent gene transcription via calcium influx [27,28]. Neuronal calcium influx depends on firing frequencies [21]. Cortical pE neurons exhibited higher bursting rates in NREMS-ON periods rather than W (Fig 1E, S1 Table), raising the possibility that somatic calcium could rise higher in NREMS than in W. To test this possibility, using in-vivo calcium imaging techniques, we investigated calcium transients in pE neurons across W and NREMS. Vigilance states were simultaneously assessed using surface EEG and neck muscle EMG recordings. Only imaging sessions were analyzed in which the animals exhibited both W and NREMS. Raw imaging frames (Fig 4A) were converted to relative fluorescence changes (ΔF/F_0_) based on the time-dependent baseline (Fig 4B) [29]. Representative ΔF/F_0_ for a frame was shown in Fig 4C and the time-series data were plotted in Fig 4D. In Fig 4D, NREMS shows larger peak ΔF/F_0_ transients than W. To quantify ΔF/F_0_ transients, the ΔF/F_0_ transient amplitude and duration for each cell were calculated as the 75th percentile of the peak ΔF/F_0_ transients and the percentage of time the ΔF/F_0_ transient exceeded 75% of each maximum. We chose the 75th percentile of the amplitude distribution rather than the maximum to avoid a single large transient determining the measurement. For pE neurons, the 75th percentiles of amplitude of ΔF/F_0_ transients were significantly higher in NREMS compared to W (Fig 4E). Similar to the amplitude of ΔF/F_0_ transients, pE neurons remained the duration of ΔF/F_0_ transients significantly longer in NREMS compared to W (Fig 4F). These results were not significantly different between cortical areas (N = 48 and 36 cells, p = 0.14, Mann-Whitney U test) and the data was pooled. Thus, our findings show that Ca^2+^ transients in pE neurons are larger and last longer in NREMS compared to W, confirming our prediction based on neuronal activity patterns across states.

**Fig 5:**
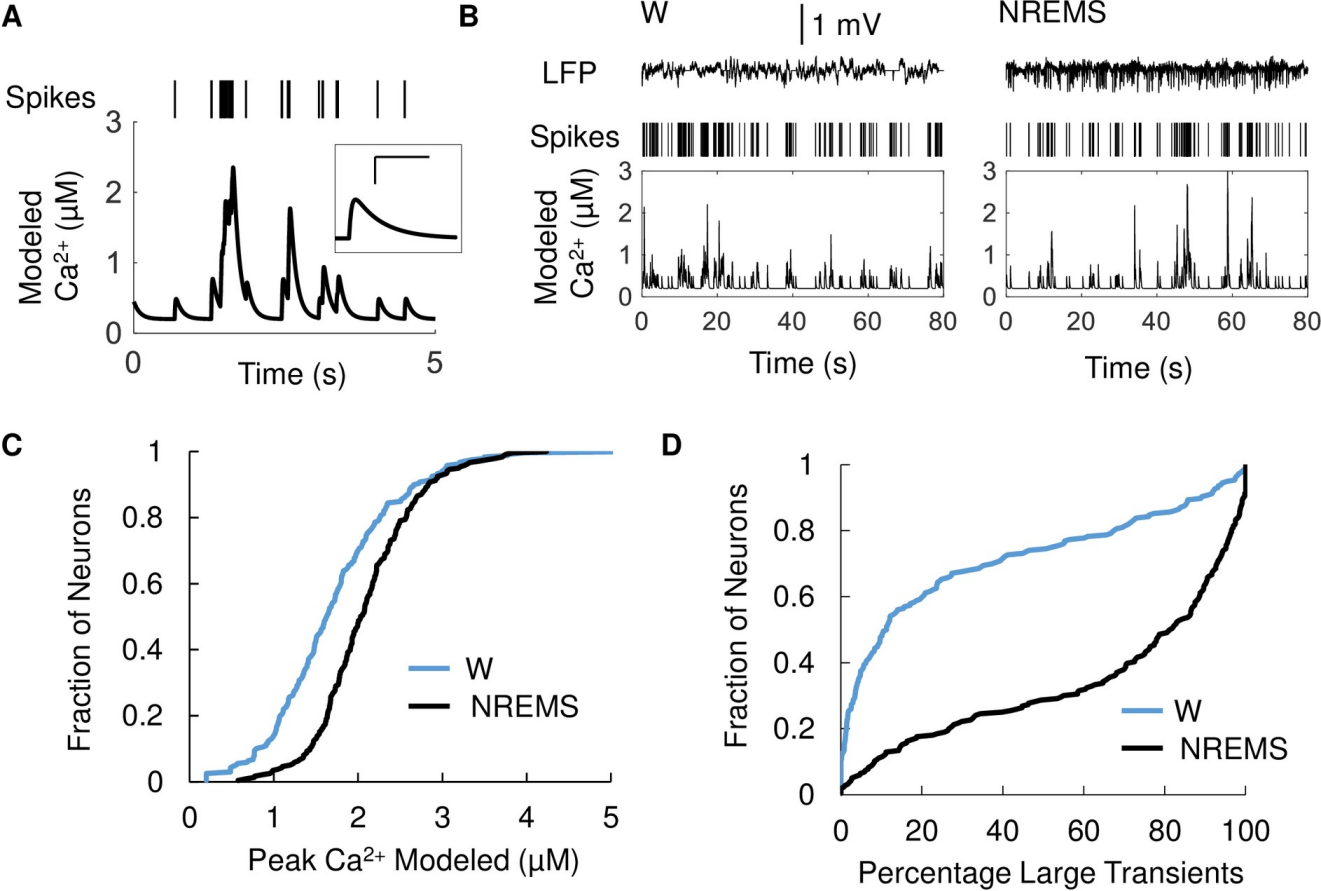
Calcium transients calculated from action potential sequences are larger in NREMS compared to W. **(A**) Example of modeled Ca^2+^ transient. Raster plot of unit activity (top) and modeled [Ca^2+^] transient (bottom). Inset: Ca^2+^ transient resulting from single action potential; scale bars 200 ms and 200 nM. **(B**) Sample W and NREMS spike trains in an M1 neuron and resulting Ca^2+^ concentrations. Local field potential (LFP, top), single unit raster plot (Spikes, middle) and modeled Ca^2+^ [Ca] (bottom) for one pE during the transition from W to NREMS. **(C**) Modeled Ca^2+^ transients were larger in NREMS compared to W. Cumulative histogram of peak Ca^2+^ modeled from single unit recordings in 217 pE in W (blue) and NREMS (black). Ca^2+^ transients were consistently larger in NREMS (2.1±0.04 μM) compared to W (1.7±0.05 μM) (p<0.01, Wilcoxon related samples signed rank test). **(D**) Majority of large Ca^2+^ transients occurred in NREMS. Cumulative Histogram of the distribution of large (top 75 percentile) Ca^2+^ peaks during either W (blue, 28.4±2.3%) or NREMS (black, 67.2±2.3%) for the same 217 pE (p<0.01, Wilcoxon related samples signed rank test).

To compare the results of our imaging study with neuronal activity data obtained in our tetrode recordings, we modeled intracellular Ca^2+^ levels from single unit activity using well established frameworks for action potential-dependent neuronal Ca^2+^ transients (Fig 5A; see Methods) [21]. The sample tetrode recordings predict larger transients in NREMS compared to W (Fig 5B). For the entirety of tetrode recordings, modeled peak Ca^2+^ concentrations, based on our observed firing rates, are larger in NREMS compared to W for pE neurons (Fig 5C). The top 75th percentile of Ca^2+^ transients occurs significantly more often in NREMS compared to W in pE neurons (Fig 5D). This result was robust over a large range of modeled decay time constants (S5 Fig).

## Discussion

Clearly, NREMS is not an inactive brain state. A significant fraction of neurons in somatosensory and motor cortex actually showed higher average activity levels in NREMS compared to waking. The higher rate of burst firing and higher peak intracellular calcium transients also indicate that energy preservation is not a primary benefit of NREMS firing at the cortical level.

Here, we show that NREMS cortical activity at the single unit level does not recapitulate W patterns [4], but represents a mode of activation distinct from W in four key aspects: first, individual entropy rate of single unit firing patterns is lower in NREMS compared to W; second, burst firing is significantly greater [9,30] and negatively correlated with individual entropy and third, intracellular peak Ca^2+^ transients are significantly larger [31]. The fourth distinction occurs at the neural network level. Spike sequences of neural ensembles are significantly less organized in NREMS compared to W (greater ensemble entropy in NREMS) and are significantly closer to random in NREMS compared to W. We don’t differentiate between active and quiet waking in our analysis—these two sub-states may show different levels of cortical organization, which future investigations may be able to resolve.

The alteration of cortical spike organization from W to NREMS we describe is compatible with networks of neurons driven by non-specific input [11]. In NREMS, these neural networks undergo alternation between high synaptic activity in the ON period and synaptic silence during the OFF period [1,32]; during NREMS-ON, the timing of action potential firing would be less determined by precise synaptic input and rely more on intracellular mechanisms. Notably, average firing is constant throughout the ON period in natural sleep, confirming earlier analysis ON-OFF firing in anesthetized animals [22,23]. Supporting evidence for cell intrinsic control of firing comes from the increased burst firing during NREMS-ON periods, which is highly dependent on the availability of certain channels such as the persistent sodium channel or low-voltage activated calcium channels [33]. As a consequence of the lower individual entropy rate, code efficiency for individual pE neurons is reduced in NREMS compared to W [17] and synchronous transitions to silent OFF periods in NREMS further reduce the code efficiency of the ensemble of neurons [17].

Ensembles of cortical neurons tend to fire in specific patterns when spontaneously active, recapitulating sequences recorded after targeted activation [11,34]. Such synfire chains reflect the inner functional structure of cortical networks, since they can be observed in vivo and in vitro [11]. Indeed, sequential structures in cortical firing during ON periods have been described [35], however, limited to the first 100 ms after ON period onset and predominantly among neurons with high firing rates, conditions that do not apply to our recordings and analysis. Nevertheless, entropies of cortical spike sequences in NREMS were lower than expected from complete randomness, indicating that the network-weighted synaptic structure is still reflected to some degree in its NREMS activity, albeit to a significantly lesser degree than W. This indicates a loss of functional connectivity, which was also observed at larger spatial scales by other authors in NREMS compared to W [8].

The activity patterns in cortical networks that we observed are compatible with a shift to a more homeostatic function of action potential firing during NREMS, since cortical neurons are more evenly contributing to overall activity.

Increased dendritic Ca^2+^ influx has been observed during spindle-rich NREMS in in-vivo recordings [31]. Unlike in our case, somatic Ca^2+^ transients were not found to be larger in these experiments. At the moment, we cannot definitively explain the cause of these differences.

The increase in Ca^2+^ influx may be due to increased synchronicity of excitatory inputs [31] during NREMS or the increased propensity for neurons to fire at higher frequencies, or both. Ca^2+^ transients calculated from action potential patterns revealed higher peaks in NREMS; therefore, at least part of the observed increase can be explained by altered firing patterns.

The exact role of the Ca^2+^ influx during NREMS needs further experiments to fully elucidate. Pharmacologically or genetically impeding Ca^2+^ influx into neurons causes reduced sleep times in mice [36], however the link between neuronal Ca^2+^ entry and sleep homeostasis is still unclear. As one of the key second messengers Ca^2+^ influx may mediate key functions of NREMS, in particular homeostatic down-scaling of synapses as postulated by the synaptic homeostasis hypothesis (SHY) [37].

## Supporting information

S1 Table Bursting of pE and pI in wake and NREMS. (DOCX)

S1 Fig Cortical pattern changes across W-NREMS transition. (DOCX)

S2 Fig Comparison of power spectrum between EEG surface electrodes and depth LFP tetrodes. (DOCX)

S3 Fig Spike frequency differences between NREMS and waking depend on wake frequency. (DOCX)

S4 Fig Manual verification of sleep scores. (DOCX)

S5 Fig Differences between modeled waking and NREMS calcium transients using different decay time constants. (DOCX)
