# Supplementary material for: Structure of cortical network activity across natural wake and sleep states in mice
Source: PLoS One. 2020 May 29;15(5):e0233561. doi: 10.1371/journal.pone.0233561 (PMC7259746; doi:10.1371/journal.pone.0233561)
Supplement: S1 Table — (DOCX) [file pone.0233561.s001.docx]

|  | **Burst Occurrence for pE and pI at Different Frequencies** | | | | | | | | | | | | |
| --- | --- | --- | --- | --- | --- | --- | --- | --- | --- | --- | --- | --- | --- |
|  |  |  |  |  |  |  |  |  |  |  |  |  |  |
|  |  |  | **Bursts per Minute Wake** | | |  | **Bursts per Minute NREMS** | | |  |  |  |  |
| Aps/Burst | F (Hz) | Type | Median | 25^th^ perc. | 75^th^ perc. |  | Median | 25^th^ perc. | 75^th^ perc. |  | N (cells) | p |  |
| 2 | 200 | pE | **1.25** | 0.75 | 3.45 |  | **2.93** | 1.36 | 5.58 |  | 212 | 4.85E-13 | ** |
| 2 | 200 | pI | **2.4** | 0.7 | 7.5 |  | **3.36** | 1.24 | 6.62 |  | 58 | 0.208 |  |
|  |  |  |  |  |  |  |  |  |  |  |  |  |  |
| 2 | 100 | pE | **3.8** | 1.36 | 8.21 |  | **6.52** | 3.72 | 11.37 |  | 212 | 1.07E-09 | ** |
| 2 | 100 | pI | **6.74** | 2.08 | 15 |  | **6.56** | 3.75 | 10.27 |  | 58 | 0.959 |  |
|  |  |  |  |  |  |  |  |  |  |  |  |  |  |
| 2 | 50 | pE | **5.84** | 1.59 | 15 |  | **9** | 5.21 | 15.05 |  | 212 | 3.65E-05 | ** |
| 2 | 50 | pI | **7.33** | 2.92 | 16.43 |  | **7.59** | 5.34 | 15.89 |  | 58 | 0.427 |  |
|  |  |  |  |  |  |  |  |  |  |  |  |  |  |
|  |  |  |  |  |  |  |  |  |  |  |  |  |  |
| 3 | 100 | pE | **0.42** | 0 | 1.52 |  | **1.24** | 0.54 | 2.5 |  | 212 | 1.02E-07 | ** |
| 3 | 100 | pI | **0.87** | 0 | 2.53 |  | **1.4** | 0.55 | 2.64 |  | 58 | 0.113 |  |
|  |  |  |  |  |  |  |  |  |  |  |  |  |  |
| 3 | 50 | pE | **1.18** | 0 | 3.58 |  | **2.51** | 1.14 | 4.68 |  | 212 | 1.84E-06 | ** |
| 3 | 50 | pI | **1.71** | 0.78 | 4.9 |  | **2.54** | 1.25 | 3.88 |  | 58 | 0.197 |  |

**Table S1**

**Bursting of pE and pI in wake and NREMS**

Burst occurrences increased in pE between wake and NREMS for all tested intra-burst frequencies (200 Hz, 100 Hz and 50 Hz) for bursts consisting of two and three action potentials. Burst firing in pI was not significantly different between wake and NREMS. Statistical evaluation by Wilcoxon related samples signed rank test.
