# Supplementary material for: Structure of cortical network activity across natural wake and sleep states in mice
Source: PLoS One. 2020 May 29;15(5):e0233561. doi: 10.1371/journal.pone.0233561 (PMC7259746; doi:10.1371/journal.pone.0233561)
Supplement: S1 Fig — (DOCX) [file pone.0233561.s002.docx]

**
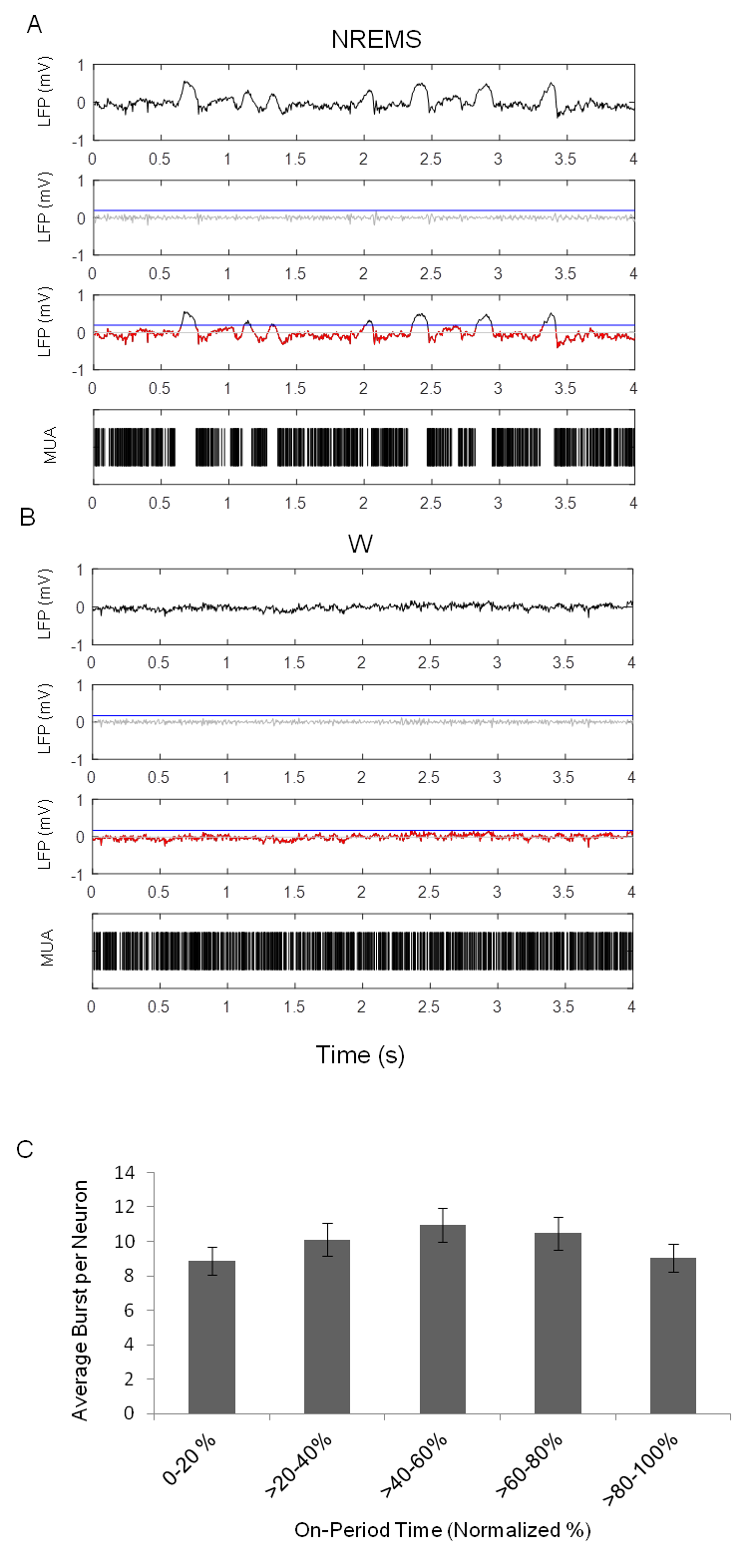
**

**Figure S1**

**Cortical pattern changes across W-NREMS transition.**

LFPs may be divided into ON and OFF periods based on the correlation of their potential relative to reference with single unit firing.

**A)** A 4 s recording from a sample tetrode during NREMS illustrates the criteria used to separate LFP positive (OFF; upward deflection) period from LFP negative (ON; downward deflection) periods. The default categorization was LFP negative (ON) period unless the threshold for LFP positive (OFF) period was exceeded. The LFP positive (OFF) period threshold was set so that spurious assignment due to noise was minimized. First, a band-pass filter was applied to the LFP (30 Hz low and 100 Hz high cutoff frequency). The mean-subtracted RMS value for the filtered LFP was calculated and a threshold set at 5 times this RMS value above the mean, resulting in a less than 3.1 10^-5^ chance of spurious threshold crossings. Row 1: unfiltered LFP. Row 2: Filtered high frequency HF-LFP in gray and 5*RMS value in blue. Row 3: Color-coded unfiltered LFP in red (negative (ON) period below threshold) and black (positive (OFF) period above threshold). Threshold in blue and mean LFP value in gray. Row 4: Simultaneous multi-unit activity from the same tetrode.

**B)** The same analysis for W from the same sample tetrode. LFP-defined positive (OFF) periods are absent and spike activity is ongoing.

**C)** Distribution of bursts (two action potentials < 15 ms apart) during the ON period. ON periods were normalized in time and values were binned at 20% intervals of the full length. There is a small but significant increase in occurrence in the middle of the ON period. (N=99 pE neurons, p=0.031, Friedman's two way analysis of variance by ranks).
