# Supplementary material for: Structure of cortical network activity across natural wake and sleep states in mice
Source: PLoS One. 2020 May 29;15(5):e0233561. doi: 10.1371/journal.pone.0233561 (PMC7259746; doi:10.1371/journal.pone.0233561)
Supplement: S2 Fig — (DOCX) [file pone.0233561.s003.docx]

**
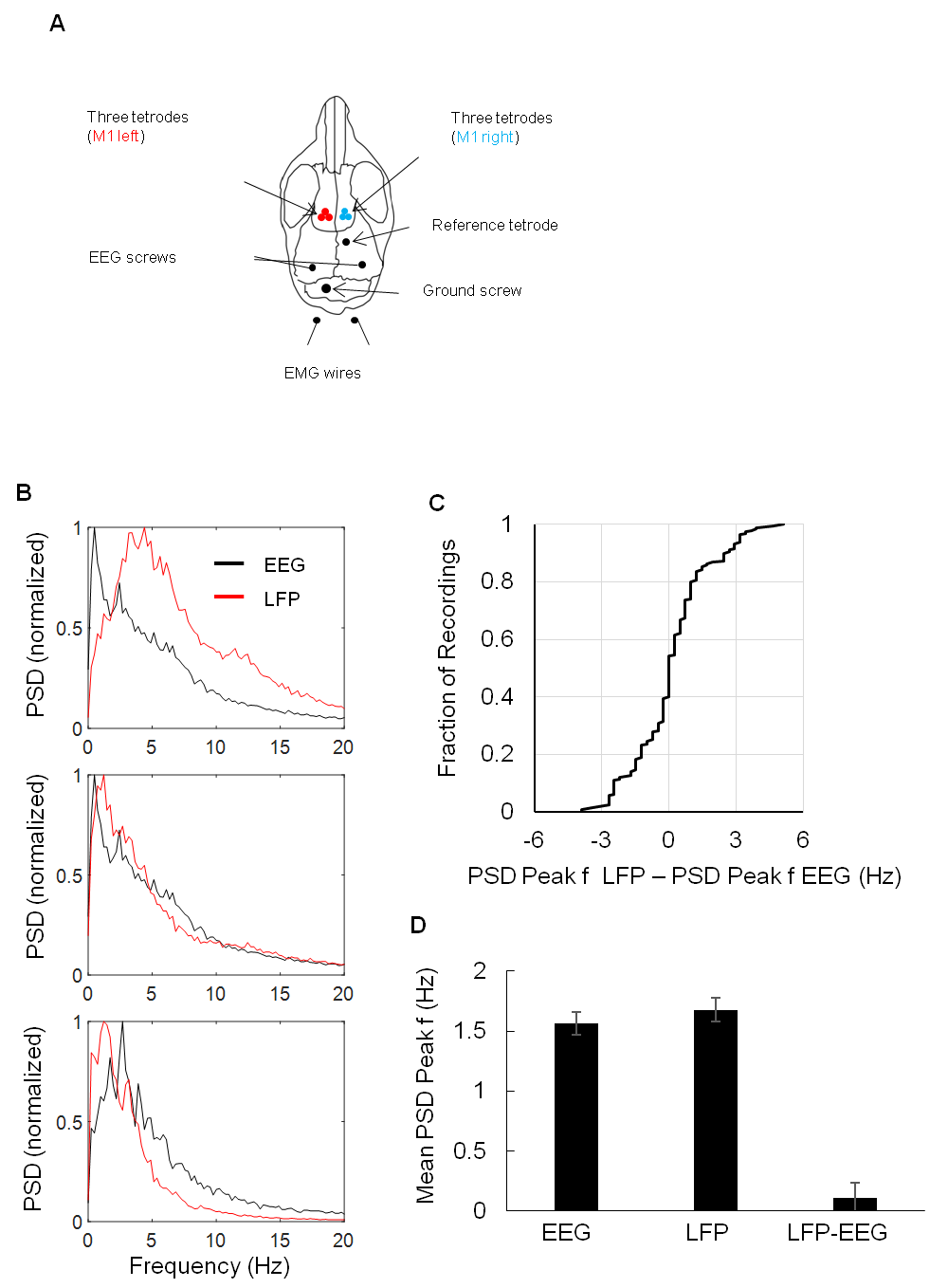
**

**Figure S2**

**Comparison of power spectrum between EEG surface electrodes and depth LFP tetrodes.**

**A)** Schematic representation of EEG, EMG and tetrode placement.

**B)** Three examples of power spectral density (PSD) plots from simultaneously recorded surface EEGs (black) and cortical tetrode LFPs (red) during NREMS. PSDs are normalized to their peak value.

**C)** Cumulative histogram of the difference in frequency at peak power between LFP and EEG for 190 simultaneous recordings (Mean difference 0.12±0.12Hz). Note the slight overhang for positive values indicating minimally higher frequencies at peak power for LFP recordings.

**D)** Summary data for PSD peak frequencies for EEGs and LFPs during NREMS (1.68±0.1 Hz LFP; 1.57±0.12Hz EEG) and the average of the pair-wise differences (p>0.48, n=190, Wilcoxon related samples signed rank test). On average, no difference in peak spectral frequency is observed between simultaneously recorded surface EEGs and depth LFPs during NREMS.
