# Supplementary material for: Structure of cortical network activity across natural wake and sleep states in mice
Source: PLoS One. 2020 May 29;15(5):e0233561. doi: 10.1371/journal.pone.0233561 (PMC7259746; doi:10.1371/journal.pone.0233561)
Supplement: S3 Fig — (DOCX) [file pone.0233561.s004.docx]

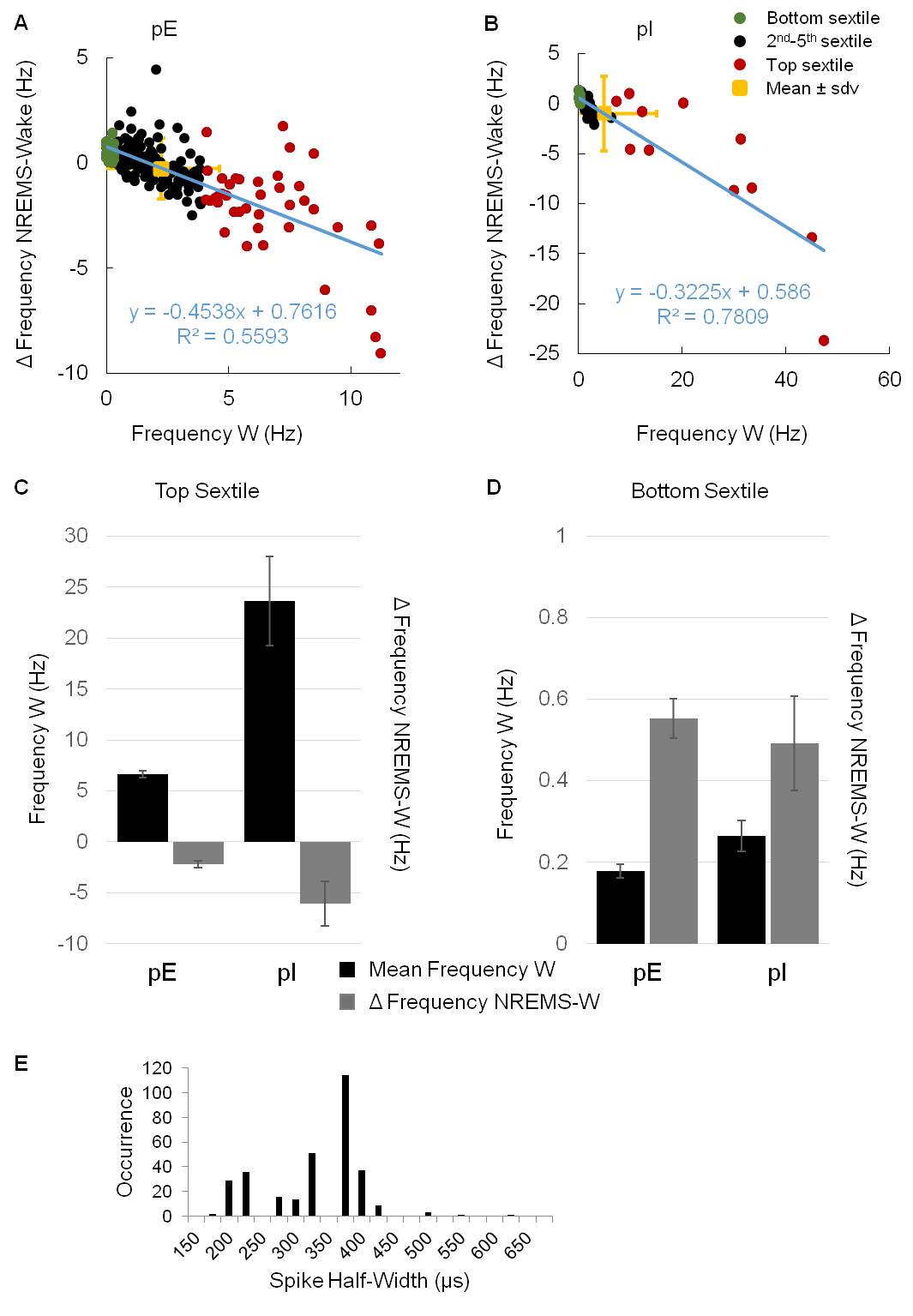


**Figure S3**

**Spike frequency differences between NREMS and waking depend on wake frequency**

Difference in average frequency for individual neurons between NREMS and waking in tetrode recordings in which neurons could be observed in both states during the same recording session. Neurons with high activity levels in waking show large reduction in average firing in NREMS. Neurons with low wake activity show increased activity during NREMS.

Overall firing rate (during both ON and OFF periods) was lower during NREMS for both pE and pI neurons, however the effect was highly non-uniform. The difference between wake and NREMS activity showed a strong dependence on wake activity, which could be fitted by a liner function with a slope of -0.45 for pE and -0.32 for pI neurons and an offset of 0.76 for pE and 0.58 for pI. Both pE and pI in their respective lowest sextile of wake activity increased firing in NREMS compared to waking; in contrast neurons in the top sextile of wake activity showed significantly decreased firing in NREMS. Individual cortical neurons thus exhibit complex alterations in firing patterns between waking and NREMS.

**A)** Change in frequency from waking to NREMS as a function of wake frequency for individual pE. Green dots mark the bottom sextile of the frequency distribution. Red dots mark the top sextile of the frequency distribution. The blue line shows a linear fit to the data, with the parameters of the fit printed in blue on the graph. Mean frequency in waking 2.23±0.15 Hz, in NREMS 1.98±0.09 Hz, n=246.

**B)** Change in frequency from waking to NREMS as a function of wake frequency for individual pI neurons. Green dots mark the bottom sextile of the frequency distribution. Red dots mark the top sextile of the frequency distribution. The blue line indicates a linear fit to the data, with the parameters of the fit printed in blue on the graph. Mean frequency in waking, 4.93±1.2 Hz, in NREMS 3.93±0.45 Hz, n=67.

**C)** Average data for the top sextile of the wake frequency distribution. Average waking frequencies in black and average change in frequency in gray. Note the decrease from wake to NREMS for both pE (N=41, -2.1±0.35 Hz, p<0.01, Wilcoxon one sample signed rank test) and pI (N=11, -6.1±2.22 Hz, p=0.021, Wilcoxon one sample signed rank test)

**D)** Average data for the bottom sextile of the wake frequency distribution. Average waking frequencies in black and average change in frequency in gray. Note the increase in average frequency from wake to NREMS for both pE (N=41, 0.55±0.05 Hz, p<0.01, Wilcoxon one sample signed rank test) and I (N=11, 0.49±0.11 Hz, p=0.04, Wilcoxon one sample signed rank test).

**E)** Distribution of spike half-width for the entire population of neurons. Neurons with values below 250 µs were classified as pI, otherwise pE.
