# Supplementary material for: Structure of cortical network activity across natural wake and sleep states in mice
Source: PLoS One. 2020 May 29;15(5):e0233561. doi: 10.1371/journal.pone.0233561 (PMC7259746; doi:10.1371/journal.pone.0233561)
Supplement: S4 Fig — (DOCX) [file pone.0233561.s005.docx]

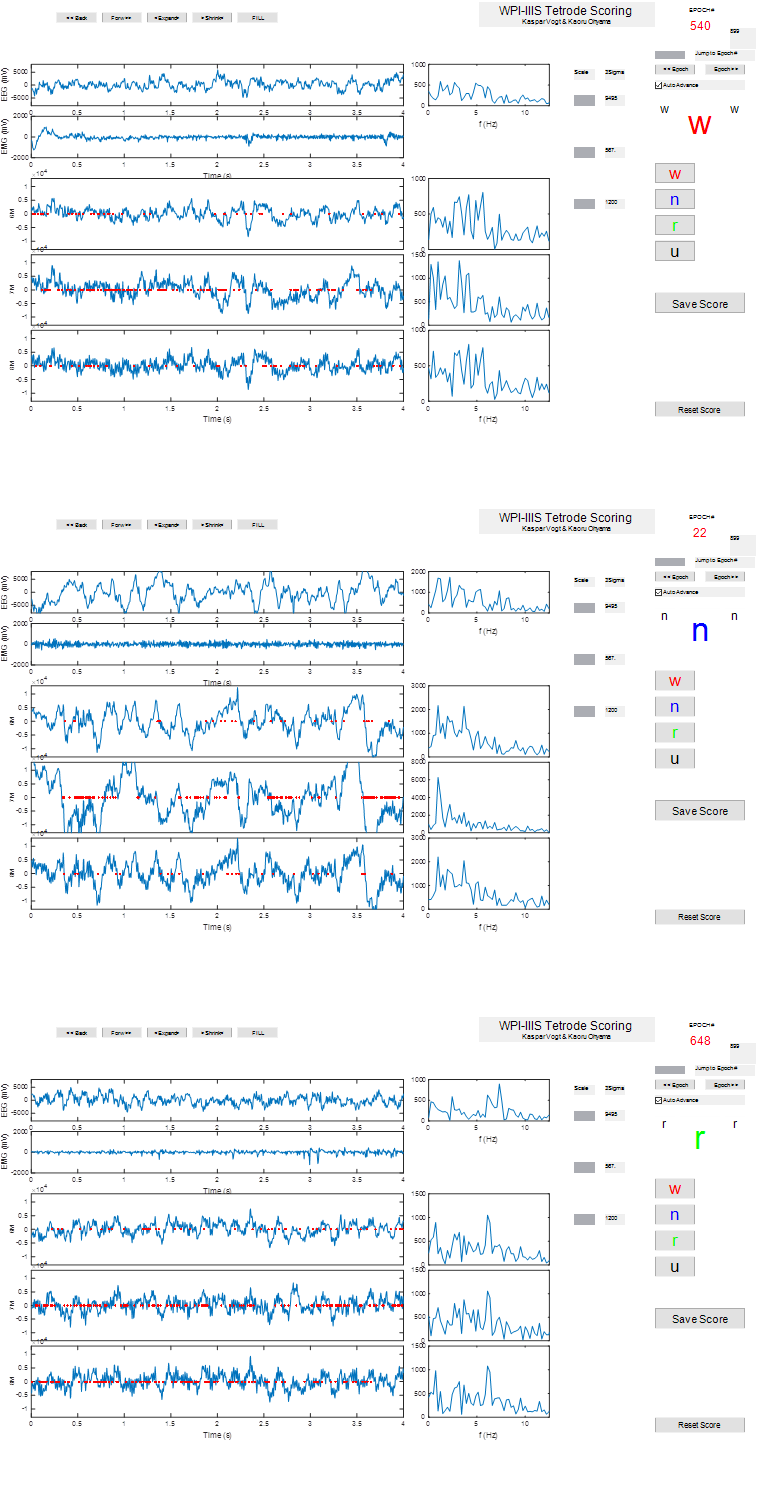


**Figure S4**

**Manual verification of sleep scores**.

Screenshots of the Matlab tool to plot EEG, EMG LFP and multi-unit activity from M1 recording; four second sample epochs for each state. Top, wake epoch with low-amplitude high-frequency EEG [10-20 Hz] and neck-motion EMG; middle, NREMS epoch with low frequency [0.5-4 Hz] high amplitude EEG and tonic activity in neck EMG; bottom, REMS epoch with low-amplitude, high-frequency EEG [10-20 Hz], with low neck muscle EMG activity.
