# Supplementary material for: Structure of cortical network activity across natural wake and sleep states in mice
Source: PLoS One. 2020 May 29;15(5):e0233561. doi: 10.1371/journal.pone.0233561 (PMC7259746; doi:10.1371/journal.pone.0233561)
Supplement: S5 Fig — (DOCX) [file pone.0233561.s006.docx]

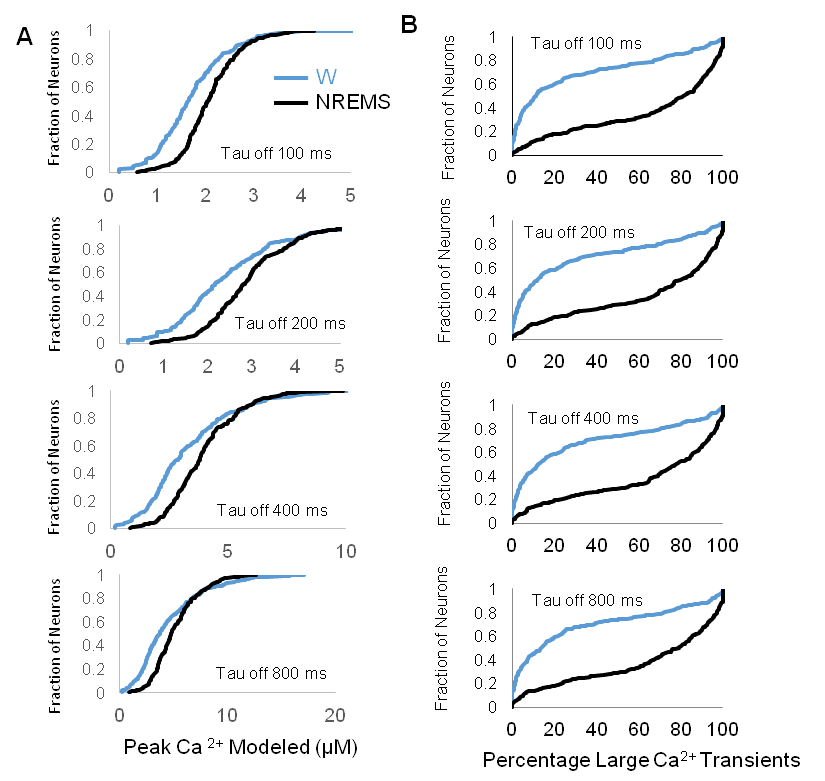


**Figure S5**

**Differences between modeled waking and NREMS Calcium transients using different decay time constants**

Calcium transients were modeled using a range of decay time constants to assess the influence of this parameter on the differences between waking and NREMS. Underlying spike data and methodology were the same as for Figure 5, except the decay time constant for the calcium transients was set to the values shown in the different panels. Statistical evaluation by Wilcoxon related samples signed rank test.

**A)** Peak 75 percentile calcium transient in waking and NREMS.

100 ms: W=1.7±0.05 µM, NREMS=2.1±0.04 µM (p<0.01)

200 ms: Waking=2.4±0.08 µM, NREMS =2.9±0.06 µM (p<0.01)

400 ms: Waking=3.3±0.12 µM, NREMS =3.9±0.09 µM (p<0.01)

800 ms Waking=4.6±0.21 µM, NREMS =5.2±0.13 µM (p<0.01)

**B)** Occurrence of the top 75^th^ percentile peaks in percentage during either waking or NREMS.

100 ms: Waking = 28.4±2.3%, NREMS = 67.2±2.3% (p<0.01)

200 ms: Waking = 28.9±2.3%, NREMS = 66.4±2.3% (p<0.01)

400 ms: Waking = 29.7±2.3%, NREMS = 65.4±2.3% (p<0.01)

800 ms: Waking = 29.7±2.4%, NREMS = 65.1±2.4% (p<0.01)
